# Supplementary material for: Genome-Wide Association Study of Body Weight Trait in Yaks
Source: Animals (Basel). 2022 Jul 21;12(14):1855. doi: 10.3390/ani12141855 (PMC9311934; doi:10.3390/ani12141855)
Supplement: Supplementary file 1 [file animals-12-01855-s001.zip › animals-1728751-supplementary.pdf]

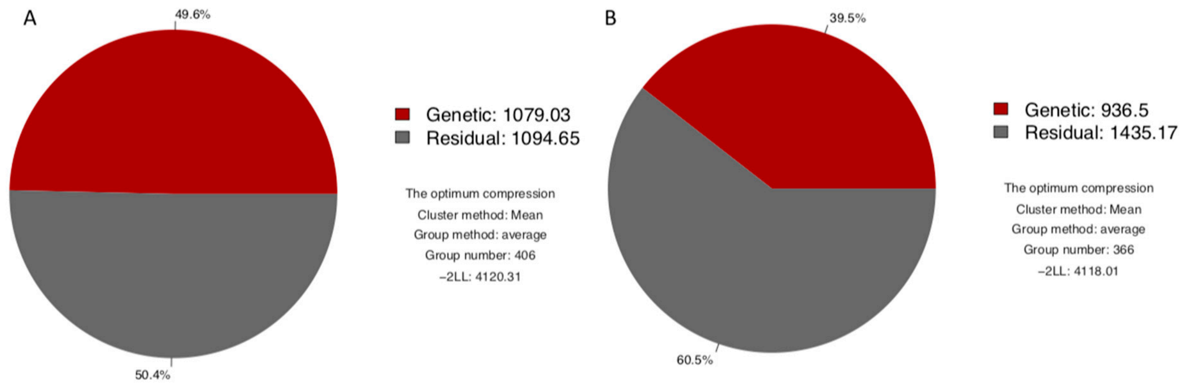

Figure S1. Genetics variance and heritability estimated.

Whole 25K markers were used to estimated additive genetics variance and heritability. The mixed linear model (MLM) got 1079.03 genetic variance, 1094.65 residual variance, and near 50% heritability in the 406 groups (individuals). The compress MLM got less genetic variance, more residual variance, and near 40% heritability in the 366 groups.

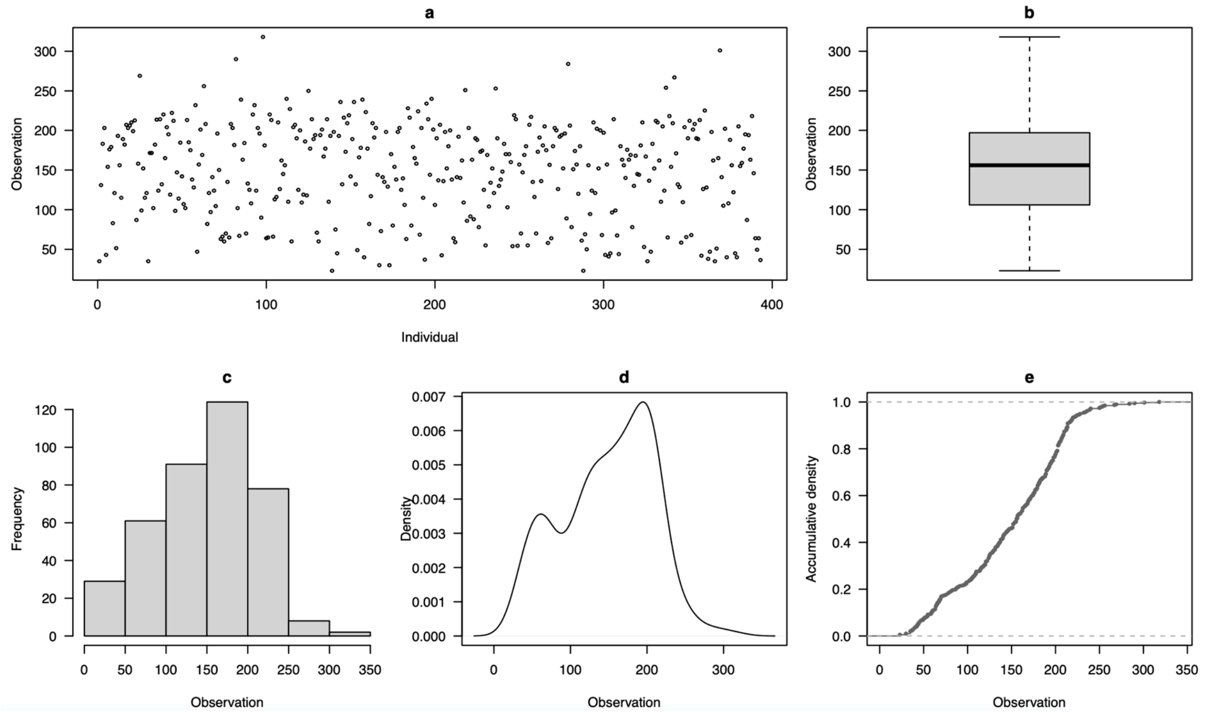

Figure S2. Phenotype values distribution after conducting with outliers.

The GAPIT function named GAPIT.Remove.outliers, was used to conduct the outliers values of phenotype. The more or less than 1.5 times IQR values were considered as outliers. The outliers values will be set equal to max or min values. The a is the phenotype point plot following individual records. The b is the box plot for phenotype after conducting with outliers. The c is the distribution plot. The d is the density plot. The e is the accumulation plot.

Table S1. Statistics of the Body Weight at different ages.

Age	Mean	Variance	Maximum	Minimum
1	88.82	1341.96	181.00	23.00
2	147.40	623.50	217.00	118.00
3	169.04	788.17	251.00	117.00
4	195.58	1798.94	301.00	130.00
5	190.95	1807.54	387.00	142.00
6	208.50	3167.47	485.00	152.00
7	210.08	1075.56	318.00	171.50
8	193.79	983.07	240.00	146.00
9	195.50	233.67	207.00	173.00
10	205.79	598.34	269.00	145.00
